# Improved PCR Performance Using Template DNA from Formalin-Fixed and Paraffin-Embedded Tissues by Overcoming PCR Inhibition

**DOI:** 10.1371/journal.pone.0077771

**Published:** 2013-10-14

**Authors:** Dimo Dietrich, Barbara Uhl, Verena Sailer, Emily Eva Holmes, Maria Jung, Sebastian Meller, Glen Kristiansen

**Affiliations:** University Hospital Bonn (UKB), Institute of Pathology, Bonn, Germany; University of Navarra, Spain

## Abstract

Formalin-fixed and paraffin-embedded (FFPE) tissues represent a valuable source for biomarker studies and clinical routine diagnostics. However, they suffer from degradation of nucleic acids due to the fixation process. Since genetic and epigenetic studies usually require PCR amplification, this degradation hampers its use significantly, impairing PCR robustness or necessitating short amplicons. In routine laboratory medicine a highly robust PCR performance is mandatory for the clinical utility of genetic and epigenetic biomarkers. Therefore, methods to improve PCR performance using DNA from FFPE tissue are highly desired and of wider interest. The effect of template DNA derived from FFPE tissues on PCR performance was investigated by means of qPCR and conventional PCR using PCR fragments of different sizes. DNA fragmentation was analyzed via agarose gel electrophoresis. This study showed that poor PCR amplification was partly caused by inhibition of the DNA polymerase by fragmented DNA from FFPE tissue and not only due to the absence of intact template molecules of sufficient integrity. This PCR inhibition was successfully minimized by increasing the polymerase concentration, dNTP concentration and PCR elongation time thereby allowing for the robust amplification of larger amplicons. This was shown for genomic template DNA as well as for bisulfite-converted template DNA required for DNA methylation analyses. In conclusion, PCR using DNA from FFPE tissue suffers from inhibition which can be alleviated by adaptation of the PCR conditions, therefore allowing for a significant improvement of PCR performance with regard to variability and the generation of larger amplicons. The presented solutions to overcome this PCR inhibition are of tremendous value for clinical chemistry and laboratory medicine.

## Introduction

Formalin-fixed, paraffin-embedded (FFPE) tissue is the most commonly used source for tissue based molecular biological testing. FFPE tissue samples are widely available, inexpensive in long term storage and in many instances they are the only available materials for retrospective studies. In the clinical routine of pathology laboratories, virtually all biopsies and surgical specimens are formalin-fixed and paraffin-embedded. Accordingly, FFPE tissue samples do not only represent a valuable source for retrospective research studies but are also the most important material for standard routine diagnostics. In recent years, several diagnostic and predictive tests have been implemented in clinical routine which are based on genotyping of somatic mutations within the tumor tissues. The accurate quantification of somatic mutations in FFPE tissue specimens is mandatory for their utility in routine diagnostics and therefore to improve clinical management of cancer in the era of personalized medicine. However, nucleic acids isolated from FFPE tissues are severely degraded and contain mainly small fragments, generally less than 300 bp. These fragments represent a poor substrate for molecular biological methods, e.g. PCR [1,2]. Furthermore, formalin-fixation leads to the formation of DNA-protein crosslinks, which are not completely removed by common lysis protocols [3]. Crosslinks increase the sensitivity of DNA to mechanical stress and decrease the accessibility for enzymes. In addition, formalin is oxidized to formic acid which causes DNA depurination and DNA strand breaks [4]. Several factors impact the extent of DNA degradation, most notably duration of fixation, type of fixative, composition of fixative (formalin concentration, pH, salt concentration), tissue type and temperature [5–7]. DNA integrity is influenced by this multitude of factors and results in a large diversity of sample quality. Highly variable or even completely ineffective target amplification is a frequently encountered problem, especially in longer DNA sequences [8]. Various studies have addressed the crucial choice of the DNA extraction method, its impact on DNA integrity and therefore on the PCR performance [5,9–11]. However, for this unresolved problem solutions allowing for robust and reliable amplification of DNA from FFPE tissue are highly desirable.

Furthermore, DNA methylation has received increasing attention in the field of personalized medicine [12–14]. DNA methylation biomarkers hold a particularly high potential for diagnostic, prognostic and predictive applications based on the analysis of tumor tissues [15]. DNA methylation in tumors is not dichotomous but rather quantitative [16]. Therefore, a highly accurate, robust and precise quantification of DNA methylation levels in FFPE tissue specimens is needed. Most methods for DNA methylation analysis rely on initial bisulfite conversion of genomic DNA [17] under harsh chemical conditions, which further increase DNA degradation [18].

In this study, the effect of DNA degradation due to formalin-fixation and paraffin-embedding on the PCR performance was investigated, using PCR assays targeting the *PITX2* and *ACTB* gene loci. These targets were chosen, because *PITX2* is a validated DNA methylation biomarker. Its methylation is strongly associated with survival of lung [19], breast [20], and prostate cancer patients [21–24]. *ACTB* is frequently used as a reference gene in DNA methylation studies [19,25–28]. Genomic and bisulfite-converted DNA was investigated in order to reveal if these findings apply to PCR-based genetic and epigenetic assays. 

## Materials and Methods

### Ethics Statement

The study has been approved by the Institutional Review Board (IRB) at the University Hospital of Bonn. The IRB waived the need for written informed consent from the participants. 

### Sample Preparation and DNA Isolation

Fourteen FFPE tissue specimens (7 cases of benign prostatic hyperplasia and 7 placental tissues collected between 1992 and 2011) were selected from the tissue archive at the Institute of Pathology, University Hospital Bonn. Unfixed human placental tissue served as source for high molecular weight (HMW) DNA. This served as reference DNA. Fifty sections (10 µm each) from each FFPE tissue specimen were deparaffinised with 25 ml xylene in a 50-ml centrifugation tube for 1 h at 37°C followed by 30 min at 60°C. After centrifugation (10 min at 4,500 g), supernatants were removed. The cell pellets were washed with 10 ml of 98% and 70% ethanol and air-dried for evaporation of residual ethanol. Lysis was carried out at 60°C for 48 h in the presence of 7 ml lysis buffer (50 mM Tris-HCl, pH 8.4, 1 mM EDTA, 0.5% [v/v] Tween®20, 0.2% [w/v] Proteinase K). DNA was purified by phenol-chloroform extraction. One volume of phenol-chloroform-isoamyl alcohol (25:24:1, pH 8) was added to the lysat and centrifuged. The upper aqueous phase was transferred into new tube, mixed with one volume of chloroform and centrifuged. In order to precipitate the DNA, one volume ice-cold isopropanol was added to the upper aqueous phase and centrifuged at 4°C. The DNA pellet was washed twice with 80% ethanol. All centrifugation steps were carried out for 10 min at 4,500 g. Air-dried DNA pellets were solubilized in 300 µl water for 1 h at 37°C. A pool of DNA from FFPE tissue from placenta and prostate, respectively, were prepared by mixing equal amounts of DNA from each specimen. The integrity of the extracted DNA was analyzed by gel electrophoresis using a 1.5% TAE agarose gel.

### DNase I Treatment

For the spiking qRT-PCR experiments, DNA from FFPE tissue was degraded with DNase I (Ambion, Invitrogene, Carlsbad, CA, USA). 71 µg DNA were incubated at 37°C over-night in the presence of 142 U DNase I. DNase I was then heat-inactivated at 95°C for 30 min. As a control, DNA was incubated in the presence of previously heat-inactivated DNase I under conditions as described above.

### Bisulfite Treatment

The bisulfite conversion and subsequent purification was performed as previously described [19] with minor modifications. In brief, 100 µl DNA was mixed with 100 µl bisulfite reagent (65% ammonium bisulfite, pH 5.3 [TIB Chemicals, Mannheim, Germany]) and 40 µl denaturation reagent (0.1 g/ml hydrochinone [Sigma-Aldrich, St. Louis, MO, USA] in DEG [diethylene glycol, Sigma-Aldrich, St. Louis, MO, USA]). The mixture was incubated for 45 min at 85°C and 1,000 rpm in a thermomixer. The converted DNA was purified by means of Nanosep centrifugal devices (10K, PALL, East Hills, NY, USA) as previously described [29].

### Endpoint PCR and qPCR

Endpoint PCR was conducted in a total volume of 20 µL containing 0.4 µM each primer (Table 1), 0.25 mM each dNTP, 1 U or 4 U FastStart *Taq* DNA polymerase (Roche, Basel, Switzerland) and 1 x FastStart *Taq* PCR reaction buffer with 2 mM MgCl_2_. Cycling conditions were at 95°C for 15 min and 40 cycles at 95°C/30 s, 54°C/30 s, 72°C/30 s. 

**Table 1:** Oligonucleotide specifications and amplicon sizes.

**Oligonucleotide**	**Amplicon Size**	**Sequence 5’ → 3’**
***PITX2* (genomic)**		
Forward primer		TTGTAACTCCTTTAAATTGATCAC
Hydrolysis probe		Cy5-CCTGAAGTGCCAGATTGGAG-BBQ-650
Reverse primers	75 bp	TCTGCATCCATTTAAAGTGTATC
	150 bp	ACTGGAAAAGAGGAACATATTTCGGA
	200 bp	ACCTTATGATAGTTTGATTTCTAA
	250 bp	TTAGTTTAGTAAGACAATACTCACACAAAA
	300 bp	AATGTATGAATTCTGATATTTACATTT
	350 bp	TGCAAGGAAATTTCTTTAAATCAGATT
	400 bp	ATTTTGTGTGATAAAAGCATCTTTAG
	450 bp	TCTTGATATTATTGGTATTTGAATTT
	500 bp	ATGTTACATCAACAAAAATCTTCAA
	550 bp	GGCTATTTAATTTTTTAGGGAAAGTTTG
	600 bp	ATTTAAGTGTAATTTTGCTGAGTT
	650 bp	GAACATTTGAAGACAAACACGCTTAAC
	700 bp	TTTTACCATGTGTATGCGTTCT
	750 bp	AGTAAGTAATTATTTAAGGAAATTAAAATACT
	850 bp	CCCATAACAAAATCTTTAATGGAAAGT
***ACTB* (genomic)**		
Forward primer		CAGGAGGCAGGGAGTATACA
Hydrolysis probe		6-FAM-TTGAAGGTTGCAGAGGCCACAG-TQ2
Reverse primer	200 bp	ACGGTACAAGGCTGTGCT
***ACTB* (bisulfite)**		
Forward primer		GTGATGGAGGAGGTTTAGTAAGTT
Hydrolysis probe		Cy5-ACCACCACCCAACACACAATAACAAACACA-BBQ-650
Reverse primer	129 bp	CCAATAAAACCTACTCCTCCCTTAA

qPCR was performed using 7500 Fast Real-Time PCR System (Applied Biosystems, Carlsbad, CA, USA). 20-µL reactions were conducted in triplicates, containing 0.4 µM of each primer (0.15 µM *ACTB* bisulfite primer, Table 1), 0.3 µM locus-specific hydrolysis probe (Table 1), 0.25 mM dNTPs, 1 x PCR buffer [35 mM Tris (pH8.4), 50 mM KCl, 6 mM MgCl_2_], 0.125 x SYBR Green® I (Lonza, Rockland, ME, USA) and varying amounts of FastStart *Taq* DNA polymerase. Cycling conditions were as follows: 95°C for 15 min initial denaturation, followed by 40 cycles consisting of annealing, extension and denaturation (95°C for 15 s). Annealing and elongation conditions were adjusted for each locus as follows: *PITX2* (genomic) locus (56°C for 1 min, 70°C for 30 s), *ACTB* (genomic) locus (58°C for 1 min, 70°C for 30 s), and *ACTB* (bisulfite-converted) locus (56°C for 45 s). The amplification under varying thermal cycling conditions was performed as followed: annealing at 56°C for 30 s, 1 min or 2 min and elongation at 70°C for 15 s, 30 s or 1 min, respectively. The threshold level was set to 0.01 and the baseline 3 to 15 for the hydrolysis probe detection. For *ACTB* bisulfite reactions default threshold level provided by Fast Real-Time PCR System were used.

qPCR employing *Pfu* polymerase was performed as followed: 20-µL reactions containing 0.6 µM of each primer (Table 1), 0.5 mM dNTPs (Roche), 1 x *Pfu* Buffer, 2.625 mM MgSO_4_, 0.125 x SYBR Green® I (Lonza) and varying amounts of recombinant *Pfu* DNA polymerase (Thermo Fisher Scientific, Waltham, MA, USA). Cycling conditions were as follows: 95°C for 3 min initial denaturation, followed by 45 cycles of 56°C for 1 min, 70°C for 30 s, 95°C for 15 s.

## Results

### PCR Amplification of DNA from FFPE Tissue under Standard PCR Conditions

**Figure 1:**
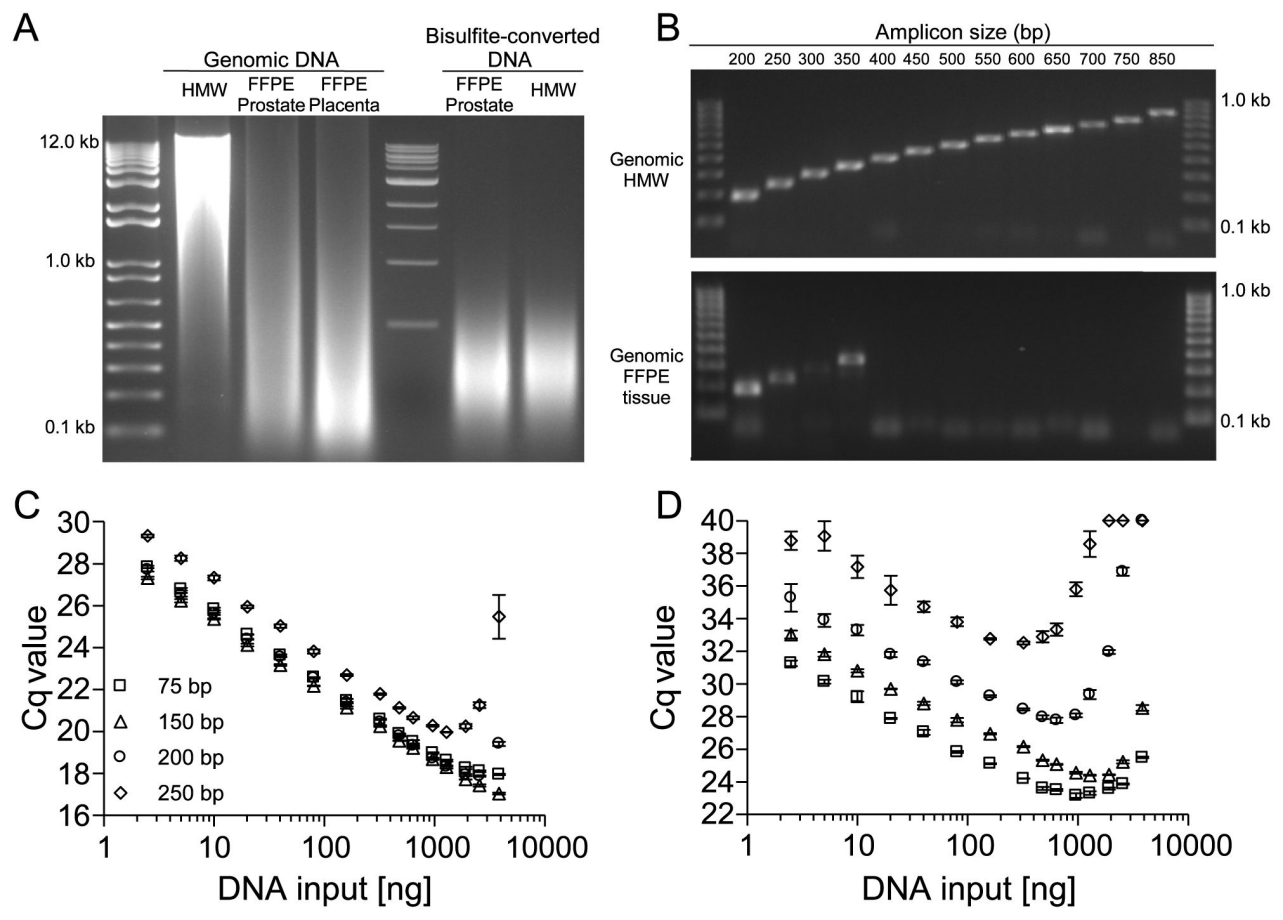
Degradation of DNA from FFPE Tissues and its Effect on PCR Amplification of Amplicons with Different Sizes. Analysis of DNA integrity of genomic and bisulfite-converted DNA from unfixed and FFPE tissues by means of (A) agarose gel electrophoresis and (B) end-point PCR using PCR fragments of different sizes within the *PITX2* gene locus. (C) qPCR results applying increasing amounts (2.5–3,840 ng) of genomic template DNA from unfixed and (D) from FFPE tissue. Shown are the mean values (± standard deviations) from triplicate measurements. Each PCR was performed with 1 U Taq polymerase. DNA from unfixed specimens is considered high molecular weight (HMW) DNA.

Isolated genomic DNA from fresh (placental) tissue predominantly contains fragments from 5 to 12 kb and therefore served as high molecular weight (HMW) DNA (Figure 1A). In contrast, genomic DNA extracted from FFPE tissue specimens (prostate and placenta) revealed a severe fragmentation resulting in fragments predominantly below 0.5 kb. The degradation is further increased due to a bisulfite conversion of the DNA as required for downstream DNA methylation analyses (Figure 1A). However, genomic DNA from FFPE tissues sporadically contained fragments of several kb length.

To assess the maximum length of successfully amplifiable PCR products using genomic template DNA from unfixed and FFPE tissues, 13 PCR fragments with lengths ranging from 200 to 850 bp targeting the *PITX2* gene locus were designed. All PCR fragments were designed using a shared forward primer and an amplicon length determining reverse primer. All PCR fragments were successfully amplified (Figure 1B, upper panel) when using genomic HMW template DNA. Fragments longer than 350 bp could not be reliably amplified when using genomic template DNA from FFPE tissue (Figure 1B, lower panel). To investigate whether this observation was due to a lack of amplifiable DNA of the required integrity, the template amount was varied and the impact on the PCR performance was analyzed. A higher input of DNA template into the PCR increases the probability that DNA fragments of sufficient length and integrity are contained in the PCR therefore enabling the amplification of larger amplicons. As shown in Figure 1C, a linear correlation of Cq values and (logarithmic) template amount was observed up to approx. 1,000 ng DNA input when applying HMW DNA. In contrast to HMW DNA, the amplification of DNA from FFPE tissue suffered from strong inhibition at higher input amounts (Figure 1D). This inhibition was indicated by increasing Cq values due to increasing DNA input amounts. PCR inhibition was highest for larger amplicons and led to a complete PCR failure (Cq=40) of the 250 bp and 200 bp fragment at high input amounts. PCR amplification with low input amounts (approx. 2.5-20 ng) showed a higher variability when amplifying DNA from FFPE tissue indicated by high standard deviation of the triplicate measurement. This high variability was most likely due to the absence of a sufficient number of template molecules with sufficient integrity. This effect obviously affected larger amplicons to a higher extent. Two opposing effects seem to impair the PCR performance: On the one hand, the lower likelihood for the presence of suited template molecules with high integrity at low input amounts. On the other hand the strong PCR inhibition due to an input of high amounts of template DNA. This resulted in a narrow range where robust PCR amplification remained feasible. In addition, this range is further narrowed with increasing amplicon size. Finally, PCR amplification of amplicons above a certain length is likely to fail completely. Accordingly, it can be hypothesized that overcoming the PCR inhibition allows for the amplification of larger amplicons since the range of robust amplification is increased. Furthermore, the input of higher amounts of template without causing inhibition would enable a more robust PCR with lower variability even for larger amplicons. 

### PCR Inhibition by Template DNA from FFPE Tissue

**Figure 2:**
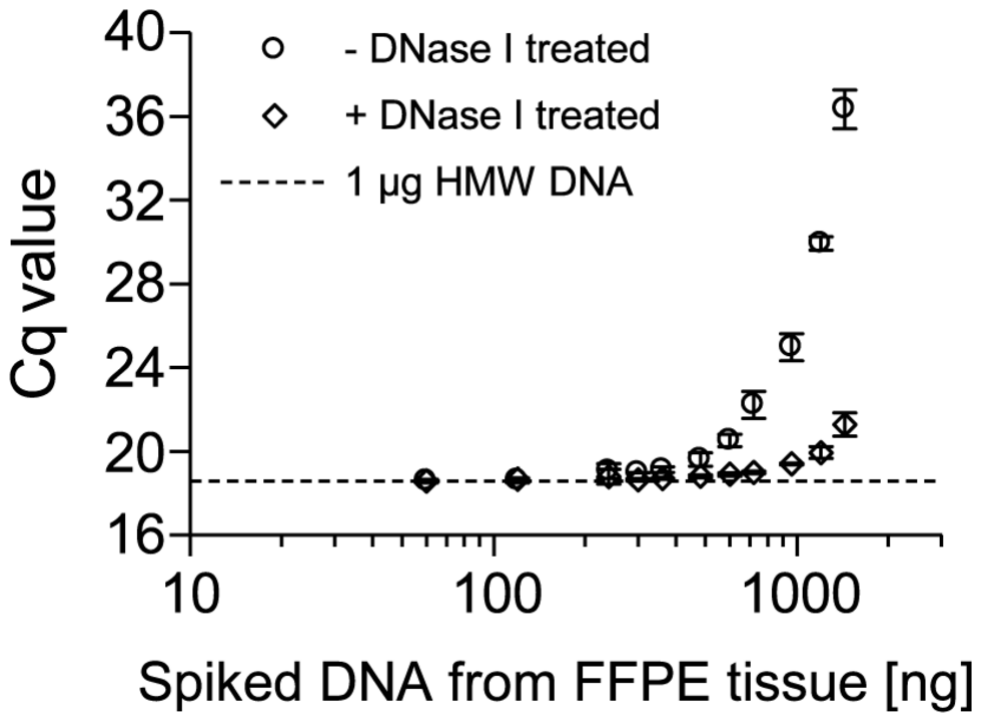
Inhibitory Effect of Template DNA from FFPE Tissues on PCR Performance. qPCR with 1 µg of genomic HMW template DNA and increasing amounts (60-1,440 ng) of spiked genomic template DNA from FFPE tissue Genomic DNA from FFPE tissue was treated beforehand with active DNase I (+) and heat-inactivated DNase I (-), respectively. qPCR was performed using a 150-bp fragment and 1 U Taq polymerase. Shown are the mean values (± standard deviations) from triplicate measurements.

To elucidate which substance contained in the extracted FFPE tissue DNA caused the inhibition, a DNA spiking experiment was performed to show whether the inhibitory effect is caused by the DNA itself or by any other substance contained in the extracted DNA. In the first case the inhibition was expected to be reduced when the DNA was enzymatically hydrolyzed. Therefore, DNA from FFPE tissue was treated with DNase I. After heat-inactivation of the DNase I increasing amounts of the hydrolyzed DNA were spiked into a qPCR which contained a constant amount of HMW DNA. The presence of HMW DNA was necessary to allow for amplification even with DNase I digested DNA from FFPE tissue which is not suited as a template anymore. For comparison, the same amounts of DNA from FFPE tissue but without prior DNase I digestion was spiked into the control arm of the experiment. The latter DNA was mixed with heat-inactivated DNase I. This allowed for a direct comparison to the DNA treated with active DNase I since it could be excluded that any residual activity of the DNase I might impair the PCR performance. As shown in Figure 2, the inhibition was significantly lower when DNase I hydrolyzed DNA from FFPE tissue was spiked-in. This result suggested that DNA from FFPE tissue itself acted as a potent PCR inhibitor. 

### Improved Amplification by Adaptation of PCR Conditions

Assuming that a PCR follows the kinetics of a regular enzyme-catalyzed reaction and DNA from FFPE tissue led to an inhibition of this bio-catalyzed reaction, an increase of the catalyst (DNA polymerase) and the reactant concentration (dNTPs) can be expected to alleviate the inhibition. A further explanation refers to an affected processivity of the polymerase. A decreased processivity (number of incorporated dNTPs before dissociation of the polymerase) would result in an amplicon-size dependent inhibition, as observed in this study. Processivity is dependent on the reaction speed. Therefore, an increased dNTP concentration might allow for the polymerization of larger DNA molecules before dissociation of the polymerase due to a higher reaction speed. In addition, an extended elongation time might allow the polymerase to re-associate with the partly polymerized DNA molecule within one PCR cycle. 

**Figure 3:**
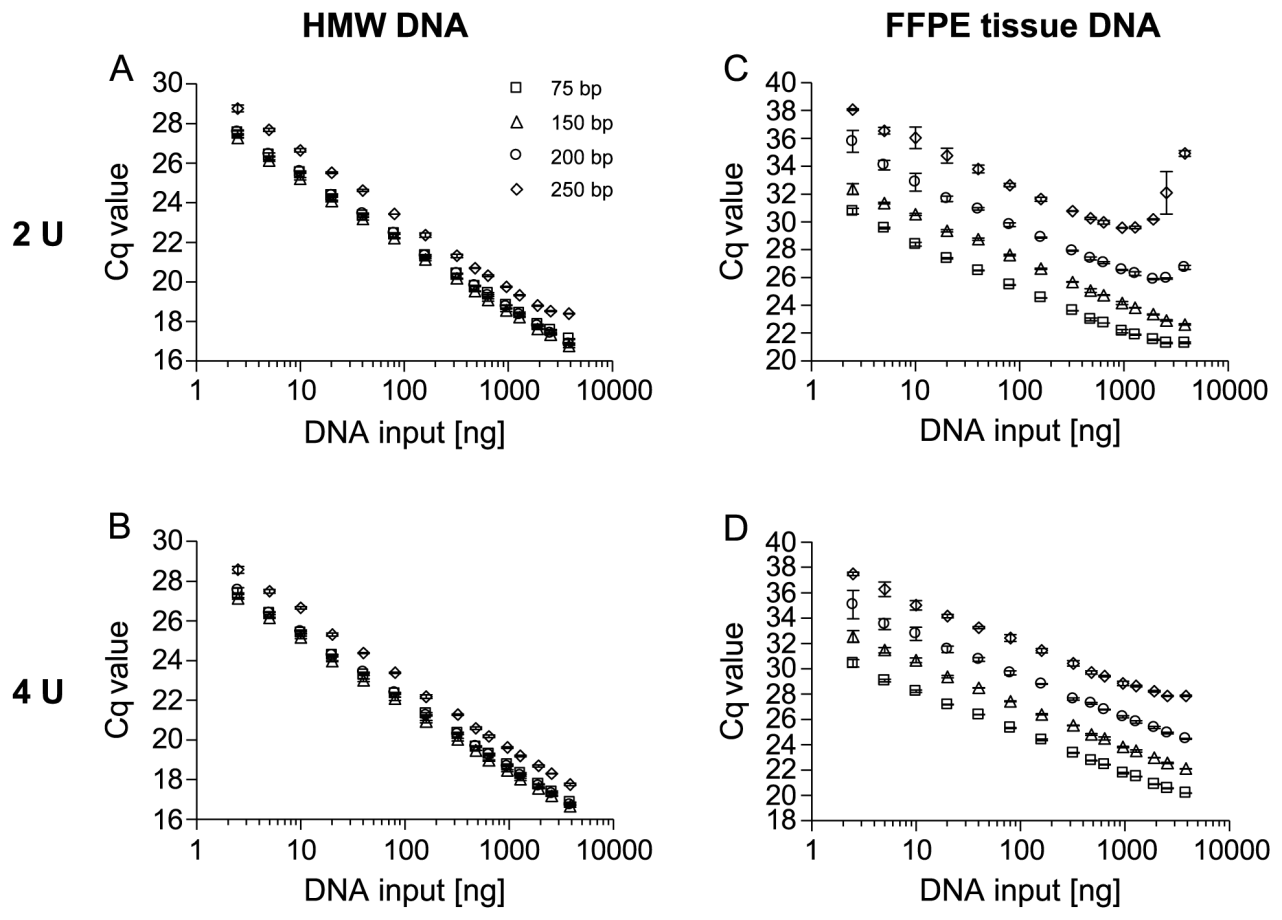
Reduction of PCR Inhibition by Increased Amounts of Taq Polymerase. qPCR with HMW and template DNA from FFPE tissue using different amounts of Taq polymerase. PCR-amplification of increasing amounts of genomic HMW template DNA (2.5-3,840 ng) using (A) 2 U *Taq*, (B) 4 U *Taq* and PCR-amplification of template DNA from FFPE tissue using (C) 2 U *Taq*, (D) 4 U *Taq*. Shown are the mean values (± standard deviations) from triplicate measurements.

In order to test these hypotheses more thoroughly, the qPCR as shown in Figure 1B-C was performed in the presence of 2 U and 4 U Taq polymerase. As shown in Figure 3A-B the amplification of HMW template DNA showed no inhibition over the whole range of DNA input amounts anymore when using 2 U and 4 U polymerase. The usage of DNA from FFPE tissue as template significantly reduced the PCR inhibition when applying 2 U and completely alleviated when using 4 U polymerase (Figure 3C-D). 

**Figure 4:**
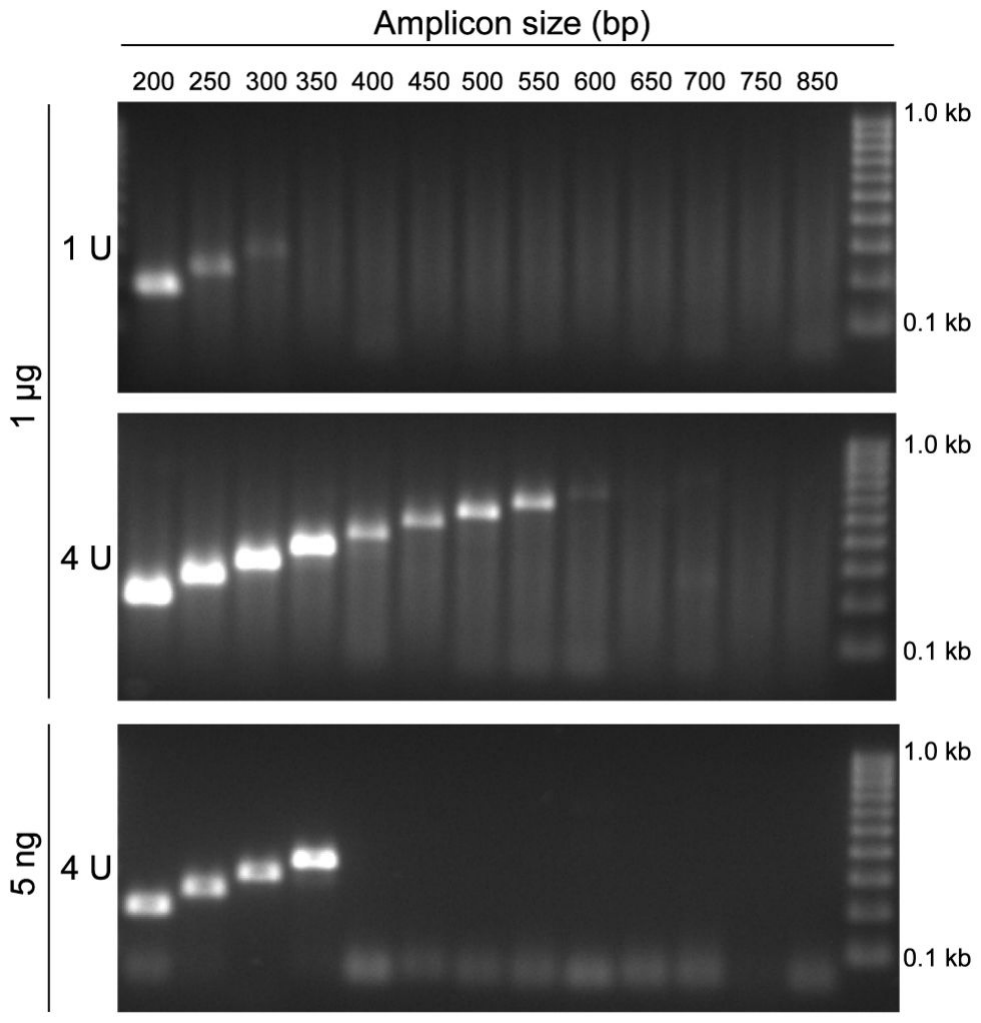
Successful PCR Amplification of Larger Fragments by Overcoming PCR Inhibition. PCR-amplified DNA fragments of different sizes within the *PITX2* gene locus using template DNA from FFPE tissue. The PCR was carried out using 1 µg (upper and middle panel) and 5 ng (lower panel) template DNA in the presence of 1 U and 4 U *Taq* DNA polymerase, respectively.

Based on these results, the experiment as shown in Figure 1B was adapted and conducted in the presence of 1 µg DNA from FFPE tissue and 1 U and 4 U Taq polymerase. The high DNA input was supposed to increase the probability for the presence of template molecules of sufficient integrity and length. Applying 1 U (Figure 4, upper panel) polymerase allowed for the successful amplification of only short amplicons up to 300 bp. The 4-fold increase of the polymerase significantly reduced PCR inhibition and allowed for the amplification of up to 600 bp fragments (Figure 4, middle panel). However, an increased polymerase concentration only resulted in a minor positive effect when using 5 ng template DNA. This was expected as only an insufficient numbers of DNA molecules were present in the reactions (Figure 4, lower panel).

**Figure 5:**
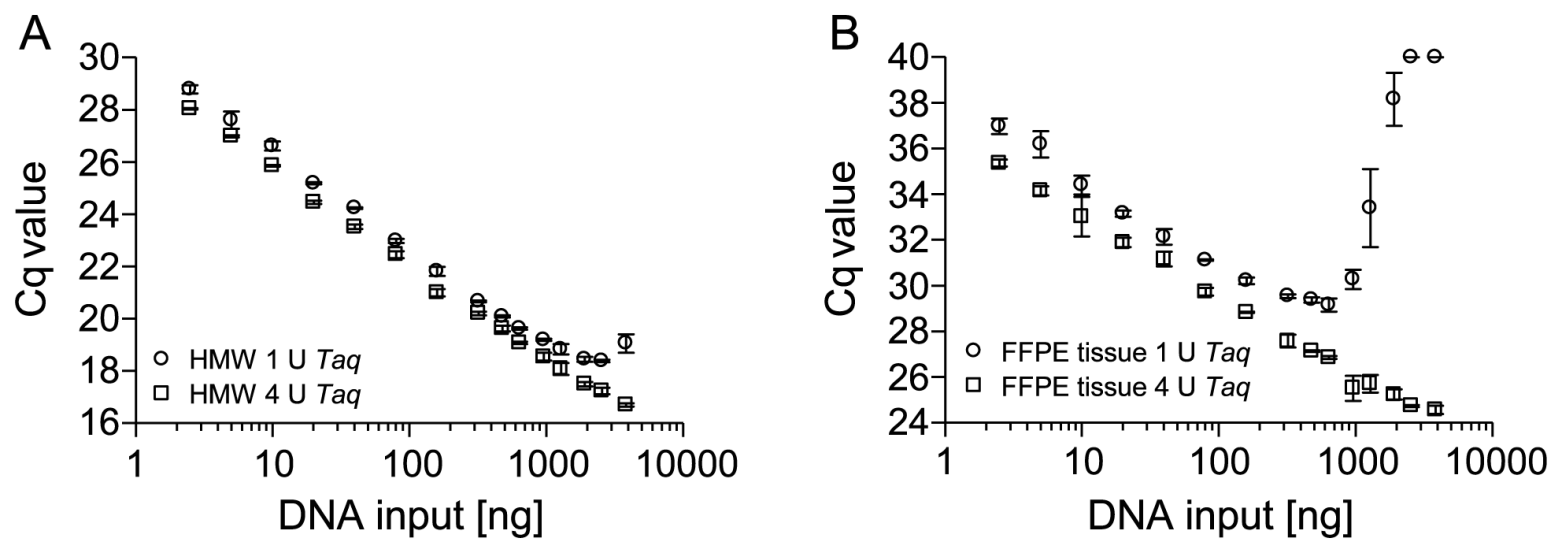
Transferability of the Findings to a qPCR Targeting an Alternative Genomic Locus. qPCR applying a 200-bp PCR fragment within the *ACTB* gene locus. Amplification with (A) HMW DNA from unfixed tissue and (B) DNA from FFPE tissue (2.5 ng to 3,840 ng) in the presence of 1 U and 4 U *Taq* DNA polymerase. Shown are the mean values (± standard deviations) from triplicate measurements.

So far, the positive effects of increased polymerase amounts on PCR efficiencies were only shown when targeting the *PITX2* gene locus and for DNA from FFPE prostate tissues. To exclude a potential locus and organ specificity of this observation, the effect of increased polymerase was also tested by qPCR targeting the *ACTB* gene locus and using template DNA from FFPE placental tissue. As shown in Figure 5, the previous findings were reproducible when using DNA from FFPE placental tissue and primers specific for the *ACTB* gene locus. PCR inhibition was completely alleviated when using 4 U Taq polymerase.

**Figure 6:**
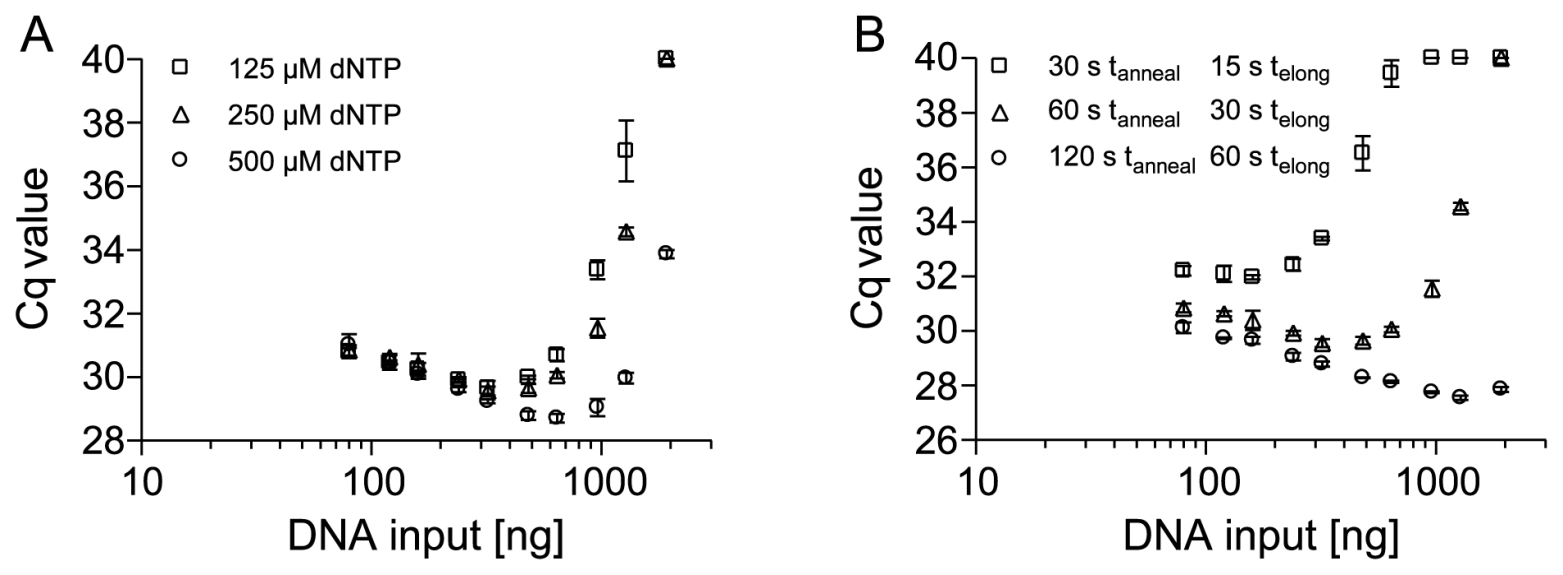
PCR Inhibition by Template DNA from FFPE Tissues with Regard to PCR Conditions (dNTP Concentration and Thermal Cycling Profile). Shown are qPCR results applying a 200-bp fragment within the *PITX2* gene locus using template DNA from FFPE tissue (80-1,920 ng) in the presence of 1 U Taq polymerase. (A) dNTP concentrations and (B) annealing and elongation times were varied. Shown are the mean values (± standard deviations) from triplicate measurements.

As described above, an increase of the dNTP concentration and an extended elongation time were expected to alleviate PCR inhibition (Figure 6). qPCR targeting a 200 bp fragment with DNA from FFPE placental tissue was performed. The halved and doubled dNTP concentrations, as compared to the standard condition, were applied. As expected, the increased dNTP concentration led to a reduction of inhibition (Figure 6A). Additionally, the usage of a prolonged annealing and extension time significantly improved the PCR performance (Figure 6B). This further supports the hypothesis that template DNA from FFPE tissue lowers the processivity of the polymerase during PCR.

### Inhibitory Effect on *Pfu* DNA Polymerase

**Figure 7:**
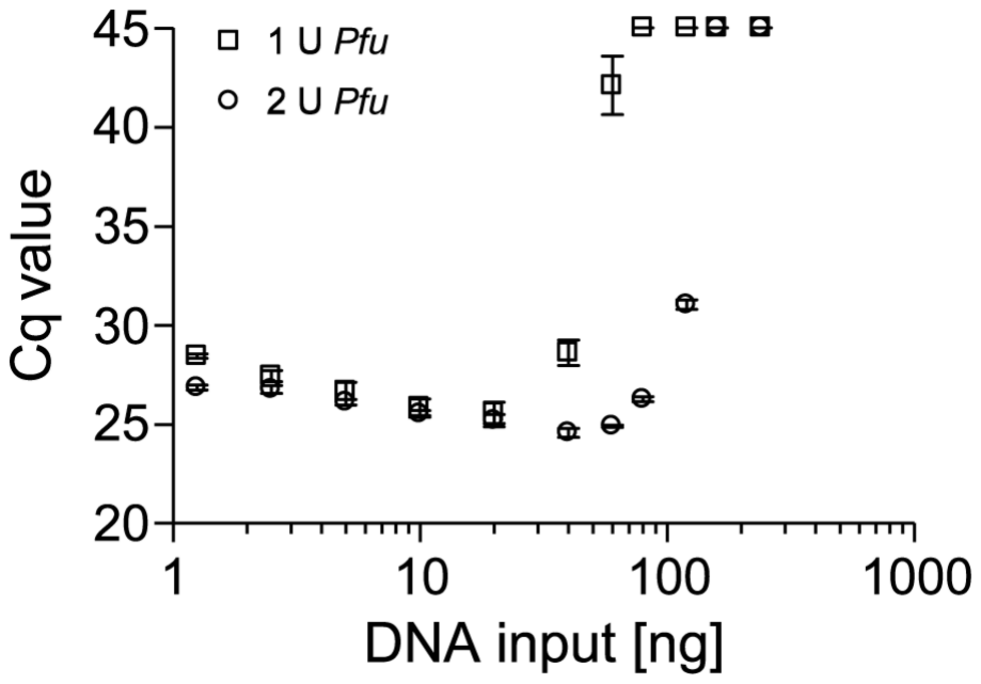
Inhibitory Effect of Template DNA from FFPE Tissues on PCR with *Pfu* Polymerase. A 75-bp PCR fragment within the *PITX2* gene locus was amplified using genomic template DNA from FFPE tissue (placenta) in the presence of 1 U and 2 U *Pfu* polymerase. Shown are the mean values (± standard deviations) from triplicate measurements.

The question remained, whether degraded DNA from FFPE tissue affects the processivity of DNA polymerases in general or if this is a phenomenon specific to polymerases from *Thermus aquaticus* (Taq polymerase). To address this question, amplification of the 75-bp amplicon was performed with *Pfu* polymerase from *Pyrococcus furiosus*. Due to its lack of 5´-3´ exonuclease activity, hydrolysis probe based qPCR was not applicable and therefore, SYBR Green was used for detection. The qPCR was performed with increasing amounts of template input (1.25–240 ng) and in the presence of 1 U and 2 U *Pfu* polymerase, respectively. As depicted in Figure 7, the previous findings from experiments with *Taq* were verified when using *Pfu* polymerase. PCR inhibition was significantly reduced by an increase of the polymerase. In the presence of 1 U *Pfu* polymerase amplification was only successful with up to 60 ng DNA from FFPE tissue. In contrast, a doubling of polymerase amount alleviated PCR inhibition and allowed a successful amplification of up to 120 ng template DNA. Interestingly, compared to the previous experiments using Taq polymerase, the inhibition already occurred at much lower input amounts when using *Pfu* polymerase. 

### Application to DNA Methylation Analyses

**Figure 8:**
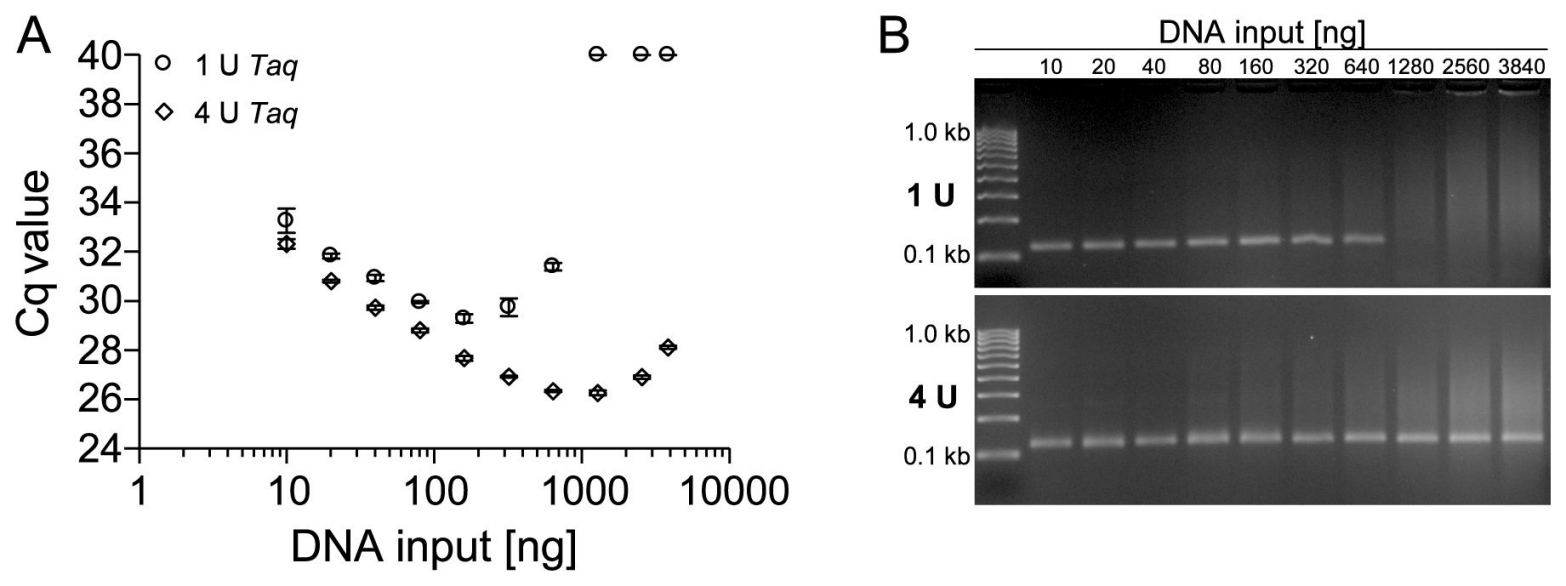
PCR Inhibition by Bisulfite-Converted Template DNA from FFPE Tissues. (A) qPCR–amplification of different amounts (10–3,840 ng) of bisulfite-converted template DNA from FFPE tissue using a 129-bp PCR fragment within the *ACTB* gene locus. 1 U and 4 U Taq polymerase were used for qPCR. Shown are the mean values (± standard deviations) from triplicate measurements. (B) PCR amplification of the specific PCR product was confirmed by agarose gel electrophoresis using 1 U (upper panel) and 4 U (lower panel) Taq polymerase.

Analyses of locus specific DNA methylation usually require a treatment of the template DNA with bisulfite to transform the epigenetic information into sequence information. As seen in Figure 1A bisulfite treatment leads to a degradation of DNA mainly to small fragments of 200 - 400 bp. To evaluate whether the findings from this study are also valid for bisulfite-converted DNA from FFPE tissue, the effect of an increased amount of Taq polymerase on PCR inhibition was studied. Therefore, primers which specifically target a bisulfite-converted sequence within the *ACTB* gene were applied. qPCR was performed using 10–3,840 ng of bisulfite-converted DNA from FFPE tissue in the presence of 1 U and 4 U Taq polymerase. As shown in Figure 8A, inhibition was significantly reduced by increasing the amount of polymerase. In the presence of 1 U Taq polymerase amplification without inhibition was only possible with an input of less than 300 ng bisulfite-converted DNA from FFPE tissue. When using 4 U Taq polymerase the amplification, with only moderate inhibition, was possible with a DNA input of up to 1,280 ng. PCR specificity was further confirmed by gel electrophoresis (Figure 8B). Therefore, adaptations of the PCR conditions, in order to improve the amplification, also apply to bisulfite-converted DNA from FFPE tissue. 

## Discussion

DNA from FFPE tissue is of tremendous importance in clinical research as well as in routine diagnostics. Alcohol-based fixatives are known to preserve biomolecules far better than crosslinking agents, i.e. formalin, but formalin-fixation ensures a higher level of morphological preservation that is needed for routine histological diagnostics. Formalin also introduces artifacts, but pathologists are highly familiar with these and the overwhelming stock of histological literature is based on formalin fixed tissues. These historical aspects in addition to its wide and inexpensive availability ensure the enduring role of formalin as the primary fixative in clinical histology. However, the increasing number of novel molecular biological tests used in the era of personalized medicine, necessitates specific requirements regarding the integrity of the tested target molecules, which may be greatly impaired by formalin-fixation. Nowadays, several diagnostic tests are available which target somatic or germ line mutations, e.g. *BRAF, KRAS, EGFR, PTEN, BRCA2, PIK3CA*, in order to personalize patients’ therapy. Most of these tests are based on a preceding amplification of the DNA via PCR. Accordingly, a robust PCR is mandatory for the application of such tests in daily clinical routine. In addition, the archives of pathological institutes harbour huge amounts of FFPE tissue specimens of patients, whose clinical follow-up data is available. These specimens represent a true treasure for clinical research and will help to elucidate the molecular mechanisms involved in carcinogenesis. Among such studies, genetic and epigenetic, i.e. DNA methylation, studies are particularly important. However, the robust and reliable PCR amplification of the highly degraded DNA derived from FFPE tissue represents a genuine challenge.

It is well accepted that DNA from FFPE tissue lacks large fragments of sufficient integrity, which allow for a robust PCR amplification, particularly of larger amplicons. However, several studies showed that DNA from FFPE tissue even contains a portion of fragments of several kb length [30,31]. Therefore, additional factors must exist which contribute to the reduced PCR performance resulting in a high variability and failed amplification of larger fragments. This failure cannot be explained by the presence of DNA-protein and DNA-DNA crosslinks, which are reported to be mainly reversible by hydrolysis especially at elevated temperatures [32–34]. In this study, it was demonstrated that DNA isolated from FFPE tissue itself exhibits an inhibitory effect on PCR leading to an instable amplification. It is shown that this inhibition affects larger amplicons to a higher extent which leads to a complete failure of amplification of fragments above a certain size. 

Until now, the mechanism by which DNA from FFPE tissue inhibits the PCR is unclear. Multiple factors are likely to play a role and further studies are needed to address this new question. The fragmentation process not only affects the target sequence and leads to an absence of template DNA, but also leads to randomly generated short DNA debris. This debris might act directly as an inhibitor of the DNA polymerase. As an enzyme inhibitor the DNA debris might bind to the polymerase and decrease its activity resulting in a decreased reaction speed. Accordingly, the inhibition can be alleviated by increasing the substrate concentration (dNTPs) or the enzyme concentration (polymerase). In addition, the prolongation of the reaction time (elongation and extension step during PCR) would allow the complete bio-polymerization of the DNA strand even at a lowered reaction speed. This is in concordance with the findings from this study that an increase of dNTP and polymerase concentration in addition to a prolonged PCR cycling helped to reduce inhibition.

Binding of an inhibitor to the polymerase might stop the substrate (dNTP) from entering the enzyme's active site and/or hinder the enzyme from catalyzing its reaction. It is likely that DNA from FFPE tissue acts as a reversible rather than an irreversible inhibitor. Reversible inhibitors bind non-covalently resulting in different types of inhibition depending on whether these inhibitors bind to the enzyme, the enzyme-substrate complex, or both. Four kinds of reversible enzyme inhibitions exist. During uncompetitive inhibition, the inhibitor binds only to the substrate-enzyme complex while during mixed inhibition, the inhibitor can bind to the enzyme as well as its substrate. However, the binding of the inhibitor affects the binding of the substrate, and vice versa. Non-competitive inhibition is a kind of mixed inhibition where the binding of the inhibitor to the enzyme reduces its activity but does not affect the binding of substrate. During competitive inhibition, the substrate is unable to bind to the enzyme. This usually results from the inhibitor having an affinity for the active site of an enzyme where the substrate would bind. Accordingly, the substrate and inhibitor compete for access to the enzyme's active site. Such competitive inhibitors are often similar in structure to the substrate. The latter type of inhibition is likely to be mainly responsible for the observed phenomenon of PCR inhibition. The small DNA fragments from FFPE tissue might either bind to the active site of the polymerase, which is responsible for binding the template DNA, or to the site which binds to the dNTPs. Clearly, more detailed kinetic studies to elucidate the type of inhibition are needed.

Another mechanism which might be considered responsible for PCR inhibition could be a reduced processivity leading to a failed amplification of larger amplicons. The DNA debris might hybridize to the template DNA, thus impairing the polymerization and leading to a dissociation of the polymerase-template complex. This would explain why *Pfu* polymerase was more affected by inhibition through DNA from FFPE tissue than Taq polymerase since *Pfu* polymerase does not show any 5’-3’ exonuclease activity and is known to exhibit a lower processivity compared with *Taq* [35]. Such exonuclease activity might help the Taq polymerase to remove DNA fragments which hybridize unspecifically to the template strand. A reduced processivity might then be balanced by an increase of the reaction speed due to an increase of dNTP and polymerase concentration. 

In summary, this study showed that polymerases from two fundamentally different sources (from the archaeum *Pyrococcus furiosus* and the bacterium *Thermus aquaticus*) are affected by PCR inhibition. Therefore, these findings seem to be valid for DNA polymerases in general. It might be possible that inhibition of reverse transcriptase is also an issue when generating cDNA from FFPE tissue extracted RNA. However, further studies are required to elucidate a potentially inhibitory effect of RNA from FFPE tissue on reverse transcriptase. In addition to PCR, other molecular biological methods which are based on DNA polymerases, i.e. nucleic acid sequence based amplification (NASBA), rolling circle amplification, and multiple displacement amplifications (MDA) which are used for whole genome amplification (WGA), might also be affected by inhibition, which ought to be analyzed in further studies. Furthermore, it was shown, that PCR inhibition also represents an issue when analyzing bisulfite-treated DNA. Finally, this study presents solutions to overcome this newly described PCR inhibition which will allow for valuable improvements in all areas of nucleic-acid based molecular biological research and diagnostics. Especially, in clinical routine diagnostics where the highly accurate diagnosis for a single patient is mandatory, template DNA is in some cases not limited and the costs for additional polymerase are negligible.
